# Supplementary material for: ZIP8 A391T Crohn’s Disease-Linked Risk Variant Induces Colonic Metal Ion Dyshomeostasis, Microbiome Compositional Shifts, and Inflammation
Source: Dig Dis Sci. 2024 Sep 25;69(10):3760–72. doi: 10.1007/s10620-024-08647-8 (PMC11489278; doi:10.1007/s10620-024-08647-8)
Supplement: Supplementary file 1 — Supplementary file1 (PDF 982 KB) [file 10620_2024_8647_MOESM1_ESM.pdf]

A

```
    G  L  A  F  G  I  L  V  G  N  N  F  A  P  N  I  I  F  A  L  A  G  G  M  F  L  Y  I  S  L  A  D  M
WT  GGACTAGCTTTCGGCATTTTGGTGGGCAACAATTTTGCTCCCAATATTATATTTGCACTCGCTGGAGGCATGTTCCTCTACATTTCTCTGGCAGATATG

    G  L  A  F  G  I  L  V  G  N  N  F  T  P  N  I  I  F  A  L  A  G  G  M  F  L  Y  I  S  L  A  D  M
mut GGACTAGCTTTCGGaATTcTcGTGGGCAACAATTTTacaCCCAAcATcATcTTTGCACTCGCTGGAGGCATGTTCCTCTACATTTCTCTGGCAGATATG

Proposed HDR sODN
GCTGTTCAACTTCCTCTCCGCGTGTTCCTGCTACGTGGGACTAGCTTTCGGaATTcTcGTGGGCAACAATTTTacaCCCAAcATcATcTTTGCACTCGCTGGAGGCA
TGTTCCTCTACATTTCTCTGGCAGATATGGTAAGAGA

sgRNA                PAM
CTAGCTTTCGGCATTTTGGT GGG
AGCGAGTGCAAATATAATAT TGG
```

B

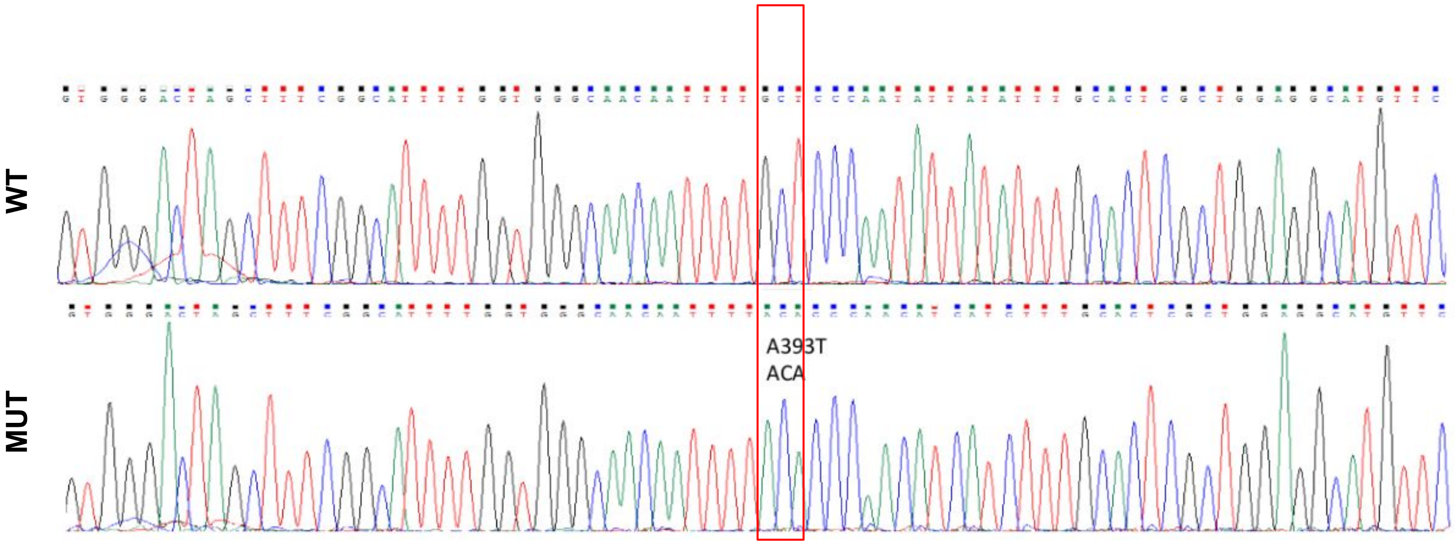

Figure S1. Gene editing strategy to generate the SLC39A8 A393T knockin line. (A) Nucleotide and corresponding amino acid sequence for the targeted sequence in SLC39A8 with **bolded and underlined** nucleotides corresponding to the 393 position. Regions highlighted yellow correspond to the regions targeted by the two guide RNA molecules (sgRNA). The single-stranded oligodeoxynucleotide sequence (sODN), used as the template for homology-directed recombination (HDR), contains the three point mutations used to generate the knockin along with additional silent mutations to prevent further Cas9-mediated mutation following HDR. The final gene-edited MUT mouse contains both the knockin and the silent mutations. (B) Sanger sequencing results demonstrate successful knockin in the gene-edited offspring.

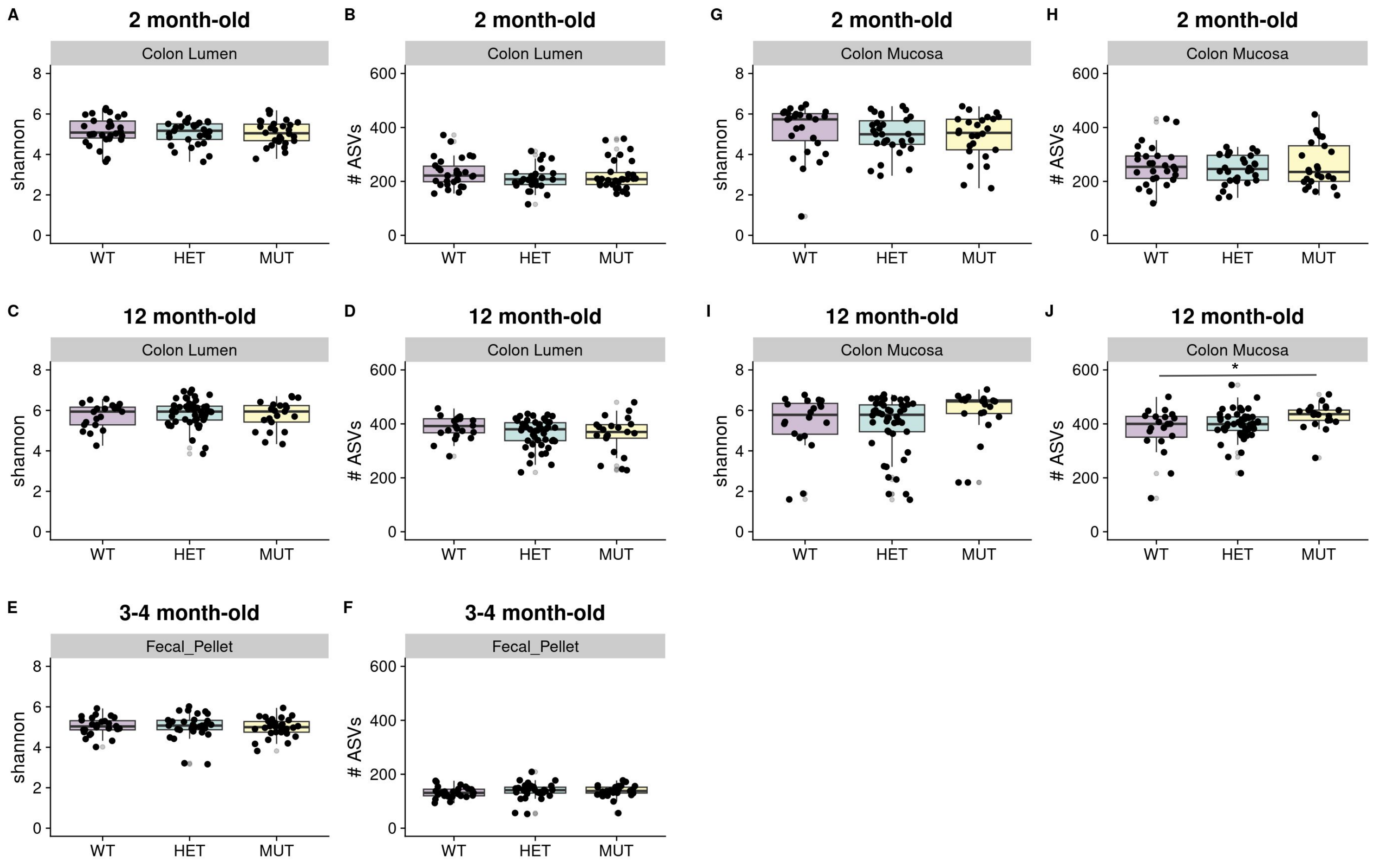

Figure S2. Minimal differences in microbiome alpha-diversity are observed between genotypes. Boxplots show the distribution of Shannon indices or the distribution of total number of amplicon sequence variants (# ASVs) across WT, HET, and MUT mice for colonic lumen datasets of 2 month-old (A, B), 12 month-old (C, D), or in the fecal pellets of 3-4 month-old mice (E, F). Distributions of Shannon index or of # ASVs are shown for the colonic mucosa datasets of 2 month-old (G, H) or 12 month-old mice (I, J). Significance of WT-HET, WT-MUT comparisons was assessed through linear mixed-effects models incorporating Sex and Genotype as fixed effects and MouseID as a random effect for the 2 month-old and 12 month-old datasets, or linear models with Sex and Genotype as covariates for the 3-4 months old dataset, *p<0.05. The data represents n=10 WT (4 F 6 M), n=10 HET (4 F 6 M), and n=10 MUT (4 F 6 M) for 2 month-old mice, and n=27 WT (11 F 16 M), n=30 HET (10 F 20 M), and n=31 MUT (14 F 17 M) for 3-4 month-old mice.

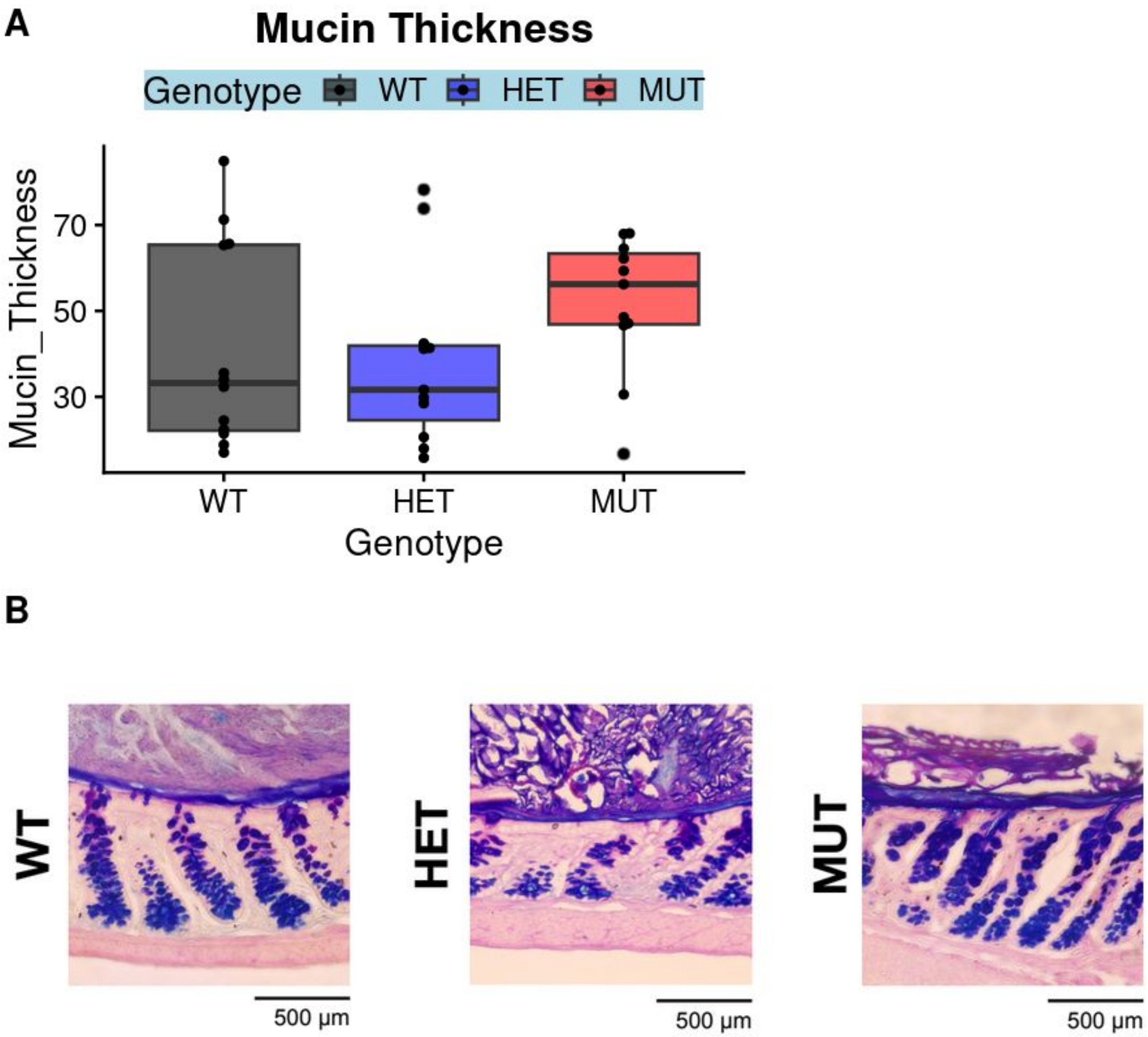

Figure S3. MUT mice do not exhibit differential mucus layer thickness compared to WT mice. Mucus layer staining of a separate cohort of 5-6 month old WT, HET, and MUT mice was achieved through PAS-Alcian Blue staining of paraffin-embedded, Carnoy's-fixed colon tissue. Mucus layer thickness measurements are shown in (A). Representative brightfield microscopy at 40X magnification of WT, HET, and MUT mucus layers are shown in (B). This data represents 5-6 month old n=12 WT (6 F 6 M), n=11 HET (5 F 6 M), and n=11 MUT (7 F 4 M) mice.

TABLE S1: 2-MONTH LUMINAL					
feature	coef	pval	qval	description	category
GOLPDLCAT.PWY	-0.2417127694	0.08617388403	0.2173051554	superpathway of glycerol degradation to 1,3-propanediol	Super-Pathways
GOLPDLCAT.PWY	-0.2417127694	0.08617388403	0.2173051554	superpathway of glycerol degradation to 1,3-propanediol	Alcohol-Degradation
P23.PWY	-0.1696159337	0.07092567432	0.186317704	reductive TCA cycle I	C1-COMPOUNDS
HOMOSER.METSYN.PWY	-0.1002579161	0.08769143883	0.2200968291	L-methionine biosynthesis I	Amino-Acid-Biosynthesis
MET.SAM.PWY	-0.09628676507	0.09189800736	0.2295186686	superpathway of S-adenosyl-L-methionine biosynthesis	Super-Pathways
PWY.5347	-0.0877745348	0.07780239477	0.199978779	superpathway of L-methionine biosynthesis (transsulfuration)	Super-Pathways
PWY.5347	-0.0877745348	0.07780239477	0.199978779	superpathway of L-methionine biosynthesis (transsulfuration)	Amino-Acid-Biosynthesis
PWY.5181	0.04332487159	0.02978705199	0.08818706779	toluene degradation III (aerobic) (via p-cresol)	AROMATIC-COMPOUNDS-DEGRADATION
PWY.5181	0.04332487159	0.02978705199	0.08818706779	toluene degradation III (aerobic) (via p-cresol)	Super-Pathways
PWY.6185	0.04362684804	0.04520969298	0.1273406352	4-methylcatechol degradation (ortho cleavage)	AROMATIC-COMPOUNDS-DEGRADATION
CRNFORCAT.PWY	0.04964005154	0.02341694094	0.07066898247	creatinine degradation I	AMINE-DEG
PWY.6944	0.05206931007	0.002833523204	0.01342613331	androstenedione degradation	Steroids-Degradation
PWY.6339	0.0542396036	0.004030040732	0.01738919703	syringate degradation	Super-Pathways
PWY.6339	0.0542396036	0.004030040732	0.01738919703	syringate degradation	AROMATIC-COMPOUNDS-DEGRADATION
METHYLGALLATE.DEGRADATION.PWY	0.0564689511	0.003739501911	0.01645267031	methylgallate degradation	AROMATIC-COMPOUNDS-DEGRADATION
GALLATE.DEGRADATION.I.PWY	0.05656847812	0.003712348129	0.01640227016	gallate degradation II	AROMATIC-COMPOUNDS-DEGRADATION
GALLATE.DEGRADATION.II.PWY	0.05656847812	0.003712348129	0.01640227016	gallate degradation I	AROMATIC-COMPOUNDS-DEGRADATION
P184.PWY	0.07060550796	0.000373150021	0.002342873818	protocatechuate degradation I (meta-cleavage pathway)	AROMATIC-COMPOUNDS-DEGRADATION
PWY.7098	0.07090143887	0.000348319907	0.002213836857	vanillin and vanillate degradation II	AROMATIC-COMPOUNDS-DEGRADATION
PWY.6338	0.07155336862	0.000388341156	0.002408427722	superpathway of vanillin and vanillate degradation	AROMATIC-COMPOUNDS-DEGRADATION
PWY.6338	0.07155336862	0.000388341156	0.002408427722	superpathway of vanillin and vanillate degradation	Super-Pathways
PWY.7097	0.07155336862	0.000388341156	0.002408427722	vanillin and vanillate degradation I	AROMATIC-COMPOUNDS-DEGRADATION
P101.PWY	0.07262945299	0.000363305913	0.002288150288	ectoine biosynthesis	Polyamine-Biosynthesis
PWY.7254	0.1137949781	0.01863060502	0.05812748765	TCA cycle VII (acetate-producers)	TCA-VARIANTS

TABLE S2: 2-MONTH MUCOSAL

feature	coef	pval	qval	description	category
PWY.6562	-0.3572907034	0.04338831714	0.1279711601	norspermidine biosynthesis	Polyamine-Biosynthesis
PWY.5180	-0.2454901222	0.07939533833	0.2020356375	toluene degradation I (aerobic) (via o-cresol)	AROMATIC-COMPOUNDS-DEGRADATION
PWY.5180	-0.2454901222	0.07939533833	0.2020356375	toluene degradation I (aerobic) (via o-cresol)	Super-Pathways
PWY.5182	-0.2454901222	0.07939533833	0.2020356375	toluene degradation II (aerobic) (via 4-methylcatechol)	AROMATIC-COMPOUNDS-DEGRADATION
PWY.5182	-0.2454901222	0.07939533833	0.2020356375	toluene degradation II (aerobic) (via 4-methylcatechol)	Super-Pathways
FAO.PWY	-0.2315183883	0.05864457784	0.1626864114	fatty acid β-oxidation I	Fatty-Acid-and-Lipid-Degradation
PWY.7094	-0.2295337934	0.05122863473	0.145378558	fatty acid salvage	Lipid-Biosynthesis
PWY.5747	-0.2222063201	0.06712086811	0.1795590102	2-methylcitrate cycle II	CARBOXYLATES-DEG
PWY0.42	-0.2164195962	0.07292118528	0.1909407595	2-methylcitrate cycle I	CARBOXYLATES-DEG
LEU.DEG2.PWY	-0.1926349302	0.06302355578	0.1709941436	L-leucine degradation I	Amino-Acid-Degradation
HSERMETANA.PWY	-0.1863974991	0.007714058653	0.0285705876	L-methionine biosynthesis III	Super-Pathways
HSERMETANA.PWY	-0.1863974991	0.007714058653	0.0285705876	L-methionine biosynthesis III	Amino-Acid-Biosynthesis
PWY.7254	-0.1825255872	0.06949279994	0.184261212	TCA cycle VII (acetate-producers)	TCA-VARIANTS
FASYN.INITIAL.PWY	-0.182079508	0.07966793885	0.2022151001	superpathway of fatty acid biosynthesis initiation (E. coli)	Super-Pathways
FASYN.INITIAL.PWY	-0.182079508	0.07966793885	0.2022151001	superpathway of fatty acid biosynthesis initiation (E. coli)	Lipid-Biosynthesis
PWY.6282	-0.1801554406	0.07847659866	0.2012949118	palmitoleate biosynthesis I (from (5Z)-dodec-5-enoate)	Lipid-Biosynthesis
PWY0.862	-0.1800522863	0.07850439508	0.2012949118	(5Z)-dodec-5-enoate biosynthesis	Lipid-Biosynthesis
PWY.5989	-0.1798270195	0.07856464518	0.2012949118	stearate biosynthesis II (bacteria and plants)	Lipid-Biosynthesis
PWY.7664	-0.1773358678	0.07719313923	0.1993918725	oleate biosynthesis IV (anaerobic)	Lipid-Biosynthesis
PWY.5920	-0.1761590811	0.04391663539	0.1289859221	superpathway of heme biosynthesis from glycine	Super-Pathways
PWY.5920	-0.1761590811	0.04391663539	0.1289859221	superpathway of heme biosynthesis from glycine	Cofactor-Biosynthesis
PWYG.321	-0.1756829287	0.07976160442	0.2022151001	mycolate biosynthesis	Lipid-Biosynthesis
P105.PWY	-0.1680747992	0.0243160698	0.07831862974	TCA cycle IV (2-oxoglutarate decarboxylase)	TCA-VARIANTS
UBISYN.PWY	-0.1674300301	0.08860575466	0.220228013	superpathway of ubiquinol-8 biosynthesis (prokaryotic)	Super-Pathways
UBISYN.PWY	-0.1674300301	0.08860575466	0.220228013	superpathway of ubiquinol-8 biosynthesis (prokaryotic)	Cofactor-Biosynthesis
PWY.5971	-0.1674273311	0.06947478374	0.184261212	palmitate biosynthesis II (bacteria and plants)	Lipid-Biosynthesis
PWY.5855	-0.1673666099	0.08915078403	0.2202548782	ubiquinol-7 biosynthesis (prokaryotic)	Cofactor-Biosynthesis
PWY.5856	-0.1673666099	0.08915078403	0.2202548782	ubiquinol-9 biosynthesis (prokaryotic)	Cofactor-Biosynthesis
PWY.5857	-0.1673666099	0.08915078403	0.2202548782	ubiquinol-10 biosynthesis (prokaryotic)	Cofactor-Biosynthesis
PWY.6708	-0.1673666099	0.08915078403	0.2202548782	ubiquinol-8 biosynthesis (prokaryotic)	Cofactor-Biosynthesis
P23.PWY	-0.1650890467	0.02053571982	0.06780662203	reductive TCA cycle I	C1-COMPOUNDS
PWY.5918	-0.1645397294	0.09081634786	0.2241061463	superpathay of heme biosynthesis from glutamate	Cofactor-Biosynthesis
PWY.5918	-0.1645397294	0.09081634786	0.2241061463	superpathay of heme biosynthesis from glutamate	Super-Pathways
PWY.6519	-0.1601092137	0.07543829173	0.1963078223	8-amino-7-oxononanoate biosynthesis I	Cofactor-Biosynthesis
PWY.5345	-0.1532502485	0.05747562041	0.1598775944	superpathway of L-methionine biosynthesis (by sulfhydrylation)	Amino-Acid-Biosynthesis
PWY.5345	-0.1532502485	0.05747562041	0.1598775944	superpathway of L-methionine biosynthesis (by sulfhydrylation)	Super-Pathways
PWY.3781	-0.1520513943	0.04722216692	0.1356587559	aerobic respiration I (cytochrome c)	Respiration
PWY.3781	-0.1520513943	0.04722216692	0.1356587559	aerobic respiration I (cytochrome c)	Electron-Transfer
BIOTIN.BIOSYNTHESIS.PWY	-0.1507405079	0.0746198649	0.1944190028	biotin biosynthesis I	Cofactor-Biosynthesis
BIOTIN.BIOSYNTHESIS.PWY	-0.1507405079	0.0746198649	0.1944190028	biotin biosynthesis I	Super-Pathways
PWY.4361	-0.1251996515	0.04507548637	0.1314701686	S-methyl-5-thio-α-D-ribose 1-phosphate degradation	Amino-Acid-Biosynthesis

PWY.4361	-0.1251996515	0.04507548637	0.1314701686	S-methyl-5-thio-α-D-ribose 1-phosphate degradation	NUCLEO-DEG
PWY.7527	-0.1238785711	0.045070561	0.1314701686	L-methionine salvage cycle III	Super-Pathways
PWY.7527	-0.1238785711	0.045070561	0.1314701686	L-methionine salvage cycle III	Amino-Acid-Biosynthesis
PWY.7211	-0.117624966	0.0574798018	0.1598775944	superpathway of pyrimidine deoxyribonucleotides de novo biosyr	Nucleotide-Biosynthesis
PWY.7211	-0.117624966	0.0574798018	0.1598775944	superpathway of pyrimidine deoxyribonucleotides de novo biosyr	Super-Pathways
PWY.7211	-0.117624966	0.0574798018	0.1598775944	superpathway of pyrimidine deoxyribonucleotides de novo biosyr	Nucleotide-Biosynthesis
MET.SAM.PWY	-0.1052499717	0.03768726316	0.1138751836	superpathway of S-adenosyl-L-methionine biosynthesis	Super-Pathways
HOMOSER.METSYN.PWY	-0.1050116684	0.04572973411	0.132852915	L-methionine biosynthesis I	Amino-Acid-Biosynthesis
PWY0.781	-0.08419247893	0.01697810603	0.05713785684	aspartate superpathway	Super-Pathways
PWY.5347	-0.08327932348	0.04823468178	0.1376261301	superpathway of L-methionine biosynthesis (transsulfuration)	Super-Pathways
PWY.5347	-0.08327932348	0.04823468178	0.1376261301	superpathway of L-methionine biosynthesis (transsulfuration)	Amino-Acid-Biosynthesis
PRPP.PWY	-0.07838320487	0.08325919669	0.2093943869	superpathway of histidine, purine, and pyrimidine biosynthesis	Super-Pathways
PWY.7539	-0.0756711268	0.09604189297	0.2339767694	6-hydroxymethyl-dihydropterin diphosphate biosynthesis III (Chla	Cofactor-Biosynthesis
P4.PWY	-0.06874926073	0.0468231943	0.1352526933	superpathway of L-lysine, L-threonine and L-methionine biosynth	Super-Pathways
P4.PWY	-0.06874926073	0.0468231943	0.1352526933	superpathway of L-lysine, L-threonine and L-methionine biosynth	Amino-Acid-Biosynthesis
PWY.7663	0.0241476503	0.0982359006	0.2373940061	gondoate biosynthesis (anaerobic)	Lipid-Biosynthesis
PWY.2942	0.02594394824	0.07731874524	0.1994709644	L-lysine biosynthesis III	Amino-Acid-Biosynthesis
PWY.5973	0.02711215681	0.08848627501	0.220228013	cis-vaccenate biosynthesis	Lipid-Biosynthesis
PWY.5097	0.02836571282	0.07810945032	0.2010169677	L-lysine biosynthesis VI	Amino-Acid-Biosynthesis
PWY.7219	0.02983416912	0.1008275238	0.2421055881	adenosine ribonucleotides de novo biosynthesis	Nucleotide-Biosynthesis
PWY.6386	0.03225171785	0.1003516908	0.2416726498	UDP-N-acetylmuramoyl-pentapeptide biosynthesis II (lysine-cont	Cell-Structure-Biosynthesis
PEPTIDOGLYCANSYN.PWY	0.03297105883	0.1031997572	0.2459926108	peptidoglycan biosynthesis I (meso-diaminopimelate containing)	Super-Pathways
PEPTIDOGLYCANSYN.PWY	0.03297105883	0.1031997572	0.2459926108	peptidoglycan biosynthesis I (meso-diaminopimelate containing)	Cell-Structure-Biosynthesis
THRESYN.PWY	0.03540821719	0.06111162697	0.1666786203	superpathway of L-threonine biosynthesis	Amino-Acid-Biosynthesis
THRESYN.PWY	0.03540821719	0.06111162697	0.1666786203	superpathway of L-threonine biosynthesis	Super-Pathways
GLYCOGENSYNTH.PWY	0.03776749024	0.09336619797	0.2285186664	glycogen biosynthesis I (from ADP-D-Glucose)	Carbohydrates-Biosynthesis
PWY.6123	0.03853258501	0.05305124528	0.1495404229	inosine-5'-phosphate biosynthesis I	Nucleotide-Biosynthesis
PWY.6121	0.03882833633	0.04252960828	0.1259692206	5-aminoimidazole ribonucleotide biosynthesis I	Nucleotide-Biosynthesis
PWY.6122	0.04246155463	0.04699095556	0.1353648926	5-aminoimidazole ribonucleotide biosynthesis II	Nucleotide-Biosynthesis
PWY.6277	0.04246155463	0.04699095556	0.1353648926	superpathway of 5-aminoimidazole ribonucleotide biosynthesis	Nucleotide-Biosynthesis
PWY.6277	0.04246155463	0.04699095556	0.1353648926	superpathway of 5-aminoimidazole ribonucleotide biosynthesis	Super-Pathways

TABLE S3: 12-MONTH LUMINAL					
feature	coef	pval	qval	description	category
PPGPPMET.PWY	-0.5508761421	0.01123100342	0.06348797464	ppGpp biosynthesis	Metabolic-Regulators
PWY0.1261	-0.1459570701	0.0357194808	0.1609823176	anhydromuropeptides recycling	SECONDARY-METABOLITE-DEGRADATION
PWY.7234	-0.1088683543	0.04312955762	0.1796154995	inosine-5'-phosphate biosynthesis III	Nucleotide-Biosynthesis
PWY.7200	-0.0869414218	0.06706238976	0.2490239981	superpathway of pyrimidine deoxyribonucleoside salvage	Nucleotide-Biosynthesis
PWY.7200	-0.0869414218	0.06706238976	0.2490239981	superpathway of pyrimidine deoxyribonucleoside salvage	Super-Pathways
FASYN.ELONG.PWY	-0.08148658986	0.02652394511	0.1272066755	fatty acid elongation -- saturated	Lipid-Biosynthesis
DENOVOPURINE2.PWY	-0.08093870563	0.0618559005	0.2366347822	superpathway of purine nucleotides de novo biosynthesis II	Super-Pathways
DENOVOPURINE2.PWY	-0.08093870563	0.0618559005	0.2366347822	superpathway of purine nucleotides de novo biosynthesis II	Nucleotide-Biosynthesis
PWY.6122	-0.0484202216	0.014599104	0.07925916199	5-aminoimidazole ribonucleotide biosynthesis II	Nucleotide-Biosynthesis
PWY.6277	-0.0484202216	0.014599104	0.07925916199	superpathway of 5-aminoimidazole ribonucleotide biosynthesis	Super-Pathways
PWY.6277	-0.0484202216	0.014599104	0.07925916199	superpathway of 5-aminoimidazole ribonucleotide biosynthesis	Nucleotide-Biosynthesis
PWY.6121	-0.04824193883	0.009817611291	0.05607628671	5-aminoimidazole ribonucleotide biosynthesis I	Nucleotide-Biosynthesis
PWY.6123	-0.04283016331	0.007582197311	0.04535532573	inosine-5'-phosphate biosynthesis I	Nucleotide-Biosynthesis
PWY.5973	-0.0368747843	0.02145622999	0.1108581466	cis-vaccenate biosynthesis	Lipid-Biosynthesis
PWY.7663	-0.03145243878	0.04225453541	0.1768669485	gondoate biosynthesis (anaerobic)	Lipid-Biosynthesis
PWY0.1061	0.2902366852	0.00392728895	0.02587997226	superpathway of L-alanine biosynthesis	Amino-Acid-Biosynthesis
CALVIN.PWY	0.03313705214	0.06201226606	0.2366825468	Calvin-Benson-Bassham cycle	C1-COMPOUNDS
PWY.1861	0.1296586209	0.0196816249	0.1031091496	formaldehyde assimilation II (RuMP Cycle)	C1-COMPOUNDS
RUMP.PWY	0.1168975016	0.01613646413	0.0870310934	formaldehyde oxidation I	C1-COMPOUNDS
CALVIN.PWY	0.03313705214	0.06201226606	0.2366825468	Calvin-Benson-Bassham cycle	Carbohydrates-Biosynthesis
FUCCAT.PWY	0.195964064	0.02148649504	0.1108581466	fucose degradation	Carbohydrates-Degradation
GLUCOSE1PMETAB.PWY	0.4829588018	0.02784290565	0.1323745081	glucose and glucose-1-phosphate degradation	Carbohydrates-Degradation
GLUCUROCAT.PWY	0.07161366127	0.05788509488	0.2235289734	superpathway of β-D-glucuronide and D-glucuronate degradation	CARBOXYLATES-DEG
PWY.5837	0.7259970898	0.008380980788	0.04866923167	1,4-dihydroxy-2-naphthoate biosynthesis I	Cofactor-Biosynthesis
PWY.5861	0.7106362482	0.008101471582	0.04742676425	superpathway of demethylmenaquinol-8 biosynthesis	Cofactor-Biosynthesis
PWY.5897	0.702184886	0.008028673755	0.04716845831	superpathway of menaquinol-11 biosynthesis	Cofactor-Biosynthesis
PWY.5898	0.702184886	0.008028673755	0.04716845831	superpathway of menaquinol-12 biosynthesis	Cofactor-Biosynthesis
PWY.5899	0.702184886	0.008028673755	0.04716845831	superpathway of menaquinol-13 biosynthesis	Cofactor-Biosynthesis
PWY.5840	0.6977595672	0.007948710223	0.04716845831	superpathway of menaquinol-7 biosynthesis	Cofactor-Biosynthesis
PWY.5838	0.6956045908	0.007906246227	0.04712237335	superpathway of menaquinol-8 biosynthesis I	Cofactor-Biosynthesis
PWY.5863	0.7248700561	0.008346371978	0.04866923167	superpathway of phylloquinol biosynthesis	Cofactor-Biosynthesis
RUMP.PWY	0.1168975016	0.01613646413	0.0870310934	formaldehyde oxidation I	Energy-Metabolism
PWY.6728	0.3453100062	0.03913292048	0.1715769693	methylaspartate cycle	Energy-Metabolism
ANAEROFRUCAT.PWY	0.02207362847	0.03969342319	0.1720825046	homolactic fermentation	Fermentation
PWY.7094	0.2652796396	0.01459166715	0.07925916199	fatty acid salvage	Lipid-Biosynthesis
SO4ASSIM.PWY	0.1423507482	0.06150767	0.2358510889	sulfate reduction I (assimilatory)	Noncarbon-Nutrients
CALVIN.PWY	0.03313705214	0.06201226606	0.2366825468	Calvin-Benson-Bassham cycle	Photosynthesis
METH.ACETATE.PWY	0.2589119533	0.02385397397	0.118908446	methanogenesis from acetate	Respiration
P562.PWY	0.2988172388	0.03165112424	0.1470793767	myo-inositol degradation I	SECONDARY-METABOLITE-DEGRADATION
GLUCUROCAT.PWY	0.07161366127	0.05788509488	0.2235289734	superpathway of β-D-glucuronide and D-glucuronate degradation	SECONDARY-METABOLITE-DEGRADATION

ANAEROFRUCAT.PWY	0.02207362847	0.03969342319	0.1720825046	homolactic fermentation	Super-Pathways
SO4ASSIM.PWY	0.1423507482	0.06150767	0.2358510889	sulfate reduction I (assimilatory)	Super-Pathways
GLUCUROCAT.PWY	0.07161366127	0.05788509488	0.2235289734	superpathway of β-D-glucuronide and D-glucuronate degradation	Super-Pathways
PWY.5861	0.7106362482	0.008101471582	0.04742676425	superpathway of demethylmenaquinol-8 biosynthesis	Super-Pathways
PWY0.1061	0.2902366852	0.00392728895	0.02587997226	superpathway of L-alanine biosynthesis	Super-Pathways
PWY.5897	0.702184886	0.008028673755	0.04716845831	superpathway of menaquinol-11 biosynthesis	Super-Pathways
PWY.5898	0.702184886	0.008028673755	0.04716845831	superpathway of menaquinol-12 biosynthesis	Super-Pathways
PWY.5899	0.702184886	0.008028673755	0.04716845831	superpathway of menaquinol-13 biosynthesis	Super-Pathways
PWY.5840	0.6977595672	0.007948710223	0.04716845831	superpathway of menaquinol-7 biosynthesis	Super-Pathways
PWY.5838	0.6956045908	0.007906246227	0.04712237335	superpathway of menaquinol-8 biosynthesis I	Super-Pathways
PWY.5863	0.7248700561	0.008346371978	0.04866923167	superpathway of phylloquinol biosynthesis	Super-Pathways

TABLE S4: 12-MONTH MUCOSAL					
feature	coef	pval	qval	description	category
PWY.7234	-0.1197205064	0.02613123826	0.07537857191	inosine-5'-phosphate biosynthesis III	Nucleotide-Biosynthesis
PWY.7184	-0.1044344694	0.05774941942	0.1573839555	pyrimidine deoxyribonucleotides de novo biosynthesis I	Nucleotide-Biosynthesis
PWY.7184	-0.1044344694	0.05774941942	0.1573839555	pyrimidine deoxyribonucleotides de novo biosynthesis I	Nucleotide-Biosynthesis
PWY.7184	-0.1044344694	0.05774941942	0.1573839555	pyrimidine deoxyribonucleotides de novo biosynthesis I	Metabolic-Clusters
PWY.2941	-0.1032063947	0.04875851293	0.1352399582	L-lysine biosynthesis II	Amino-Acid-Biosynthesis
PWY.7197	-0.1014718125	0.06272105842	0.1696998334	pyrimidine deoxyribonucleotide phosphorylation	Nucleotide-Biosynthesis
PWY.7197	-0.1014718125	0.06272105842	0.1696998334	pyrimidine deoxyribonucleotide phosphorylation	Metabolic-Clusters
PWY.7196	-0.08804402921	0.06122986707	0.1661447189	superpathway of pyrimidine ribonucleosides salvage	Nucleotide-Biosynthesis
PWY.7196	-0.08804402921	0.06122986707	0.1661447189	superpathway of pyrimidine ribonucleosides salvage	Super-Pathways
PWY.7228	-0.08764367227	0.07218540504	0.1936303783	superpathway of guanosine nucleotides de novo biosynthesis I	Super-Pathways
PWY.7228	-0.08764367227	0.07218540504	0.1936303783	superpathway of guanosine nucleotides de novo biosynthesis I	Nucleotide-Biosynthesis
PWY.6125	-0.08139965848	0.07297719314	0.1954746245	superpathway of guanosine nucleotides de novo biosynthesis II	Super-Pathways
PWY.6125	-0.08139965848	0.07297719314	0.1954746245	superpathway of guanosine nucleotides de novo biosynthesis II	Nucleotide-Biosynthesis
PWY.7200	-0.07755808087	0.07406579578	0.1978253093	superpathway of pyrimidine deoxyribonucleoside salvage	Nucleotide-Biosynthesis
PWY.7200	-0.07755808087	0.07406579578	0.1978253093	superpathway of pyrimidine deoxyribonucleoside salvage	Super-Pathways
PWY0.162	-0.07624519076	0.05802992895	0.1579188923	superpathway of pyrimidine ribonucleotides de novo biosynthesis	Nucleotide-Biosynthesis
PWY0.162	-0.07624519076	0.05802992895	0.1579188923	superpathway of pyrimidine ribonucleotides de novo biosynthesis	Super-Pathways
PWY0.166	-0.07503903496	0.09701472218	0.2488407717	superpathway of pyrimidine deoxyribonucleotides de novo biosynthesis (E. coli)	Nucleotide-Biosynthesis
PWY0.166	-0.07503903496	0.09701472218	0.2488407717	superpathway of pyrimidine deoxyribonucleotides de novo biosynthesis (E. coli)	Super-Pathways
PWY0.166	-0.07503903496	0.09701472218	0.2488407717	superpathway of pyrimidine deoxyribonucleotides de novo biosynthesis (E. coli)	Nucleotide-Biosynthesis
PWY.7187	-0.07496492223	0.08732852726	0.2277343374	pyrimidine deoxyribonucleotides de novo biosynthesis II	Nucleotide-Biosynthesis
PWY.7187	-0.07496492223	0.08732852726	0.2277343374	pyrimidine deoxyribonucleotides de novo biosynthesis II	Nucleotide-Biosynthesis
DENOVOPURINE2.PWY	-0.07070018531	0.05643161779	0.1542409378	superpathway of purine nucleotides de novo biosynthesis II	Super-Pathways
DENOVOPURINE2.PWY	-0.07070018531	0.05643161779	0.1542409378	superpathway of purine nucleotides de novo biosynthesis II	Nucleotide-Biosynthesis
PWY.841	-0.07042416458	0.05917639493	0.160805421	superpathway of purine nucleotides de novo biosynthesis I	Nucleotide-Biosynthesis
PWY.841	-0.07042416458	0.05917639493	0.160805421	superpathway of purine nucleotides de novo biosynthesis I	Super-Pathways
FASYN.ELONG.PWY	-0.07018233925	0.04863063751	0.1350851042	fatty acid elongation -- saturated	Lipid-Biosynthesis
PWY.5088	0.4500994296	0.02901318937	0.0831800154	L-glutamate degradation VIII (to propanoate)	Amino-Acid-Degradation
PWY.1882	0.3392060786	0.08320947032	0.2175979872	superpathway of C1 compounds oxidation to CO2	C1-COMPOUNDS
PWY.6749	0.3259263014	0.04440767684	0.1235376767	CMP-legionaminate biosynthesis I	Carbohydrates-Biosynthesis
PWY.6897	0.04865136887	0.07893880935	0.2084880187	thiamin salvage II	Cofactor-Biosynthesis
FOLSYN.PWY	0.05245942723	0.02413390877	0.07004810982	superpathway of tetrahydrofolate biosynthesis and salvage	Cofactor-Biosynthesis
PWY.6612	0.0691851483	0.04242079623	0.1188923661	superpathway of tetrahydrofolate biosynthesis	Cofactor-Biosynthesis
PWY.7377	0.4925770223	0.09455151804	0.2435221103	cob(II)yrinate a,c-diamide biosynthesis I (early cobalt insertion)	Cofactor-Biosynthesis
PWY.5507	0.6496178914	0.02358443438	0.06855940227	adenosylcobalamin biosynthesis I (early cobalt insertion)	Cofactor-Biosynthesis
PWY.5741	0.5453314262	0.008032035721	0.02448791378	ethylmalonyl-CoA pathway	Energy-Metabolism
PWY.6728	1.374024239	0.001335409665	0.00436218314	methylaspartate cycle	Energy-Metabolism
PWY.5088	0.4500994296	0.02901318937	0.0831800154	L-glutamate degradation VIII (to propanoate)	Fermentation
METH.ACETATE.PWY	0.2784705787	0.08843731799	0.2293498911	methanogenesis from acetate	Respiration
PWY.7237	0.2137000538	0.04331254576	0.1212104825	myo-, chiro- and scillo-inositol degradation	SECONDARY-METABOLITE-DEGRADATION

P562.PWY	0.3332018527	0.01677014965	0.04983206115	myo-inositol degradation I	SECONDARY-METABOLITE-DEGRADATION
PWY.6897	0.04865136887	0.07893880935	0.2084880187	thiamin salvage II	Super-Pathways
FOLSYN.PWY	0.05245942723	0.02413390877	0.07004810982	superpathway of tetrahydrofolate biosynthesis and salvage	Super-Pathways
PWY.6612	0.0691851483	0.04242079623	0.1188923661	superpathway of tetrahydrofolate biosynthesis	Super-Pathways
PWY.7237	0.2137000538	0.04331254576	0.1212104825	myo-, chiro- and scillo-inositol degradation	Super-Pathways
PWY.1882	0.3392060786	0.08320947032	0.2175979872	superpathway of C1 compounds oxidation to CO2	Super-Pathways
PWY.5088	0.4500994296	0.02901318937	0.0831800154	L-glutamate degradation VIII (to propanoate)	Super-Pathways
PWY.5507	0.6496178914	0.02358443438	0.06855940227	adenosylcobalamin biosynthesis I (early cobalt insertion)	Super-Pathways

TABLE S5: 3-4 MONTH FECAL PELLETS					
feature	coef	pval	qval	description	category
GOLPDLCAT.PWY	-0.1769427398	0.0149836236	0.1756881841	superpathway of glycerol degradation to 1,3-propanediol	Alcohol-Degradation
GLUTORN.PWY	-0.04645535177	0.04191150945	0.2496335161	L-ornithine biosynthesis	Amino-Acid-Biosynthesis
COLANSYN.PWY	-0.04415063059	0.01751043647	0.1756881841	colanic acid building blocks biosynthesis	Carbohydrates-Biosynthesis
PWY.1269	-0.06044474317	0.03545778741	0.2290113368	CMP-3-deoxy-D-manno-octulosonate biosynthesis I	Carbohydrates-Biosynthesis
PWY.7323	-0.04961117381	0.01961060837	0.1756881841	superpathway of GDP-mannose-derived O-antigen building blocks biosynthesis	Carbohydrates-Biosynthesis
PWY.1269	-0.06044474317	0.03545778741	0.2290113368	CMP-3-deoxy-D-manno-octulosonate biosynthesis I	Carbohydrates-Biosynthesis
PWY.6901	-0.06323344154	0.02041669144	0.1756881841	superpathway of glucose and xylose degradation	Carbohydrates-Degradation
PWY.5384	-0.1318414954	0.009895895383	0.141667555	sucrose degradation IV (sucrose phosphorylase)	Carbohydrates-Degradation
GLUCUROCAT.PWY	-0.05970682386	0.005041720407	0.09700305131	superpathway of β-D-glucuronide and D-glucuronate degradation	CARBOXYLATES-DEG
NAGLIPASYN.PWY	-0.06151006075	0.03159354502	0.2130606011	lipid IVA biosynthesis	Cell-Structure-Biosynthesis
PWY.6467	-0.06110900162	0.03241046356	0.2150157583	Kdo transfer to lipid IVA III (Chlamydia)	Cell-Structure-Biosynthesis
PWY.7323	-0.04961117381	0.01961060837	0.1756881841	superpathway of GDP-mannose-derived O-antigen building blocks biosynthesis	Cell-Structure-Biosynthesis
PANTOSYN.PWY	-0.04086930306	0.004019143793	0.09595114474	pantothenate and coenzyme A biosynthesis I	Cofactor-Biosynthesis
PANTOSYN.PWY	-0.04086930306	0.004019143793	0.09595114474	pantothenate and coenzyme A biosynthesis I	Cofactor-Biosynthesis
PWY.6897	-0.06270223199	0.001512684015	0.0726088327	thiamin salvage II	Cofactor-Biosynthesis
PWY.7539	-0.05668053461	0.0051116804	0.09700305131	6-hydroxymethyl-dihydropterin diphosphate biosynthesis III (Chlamydia)	Cofactor-Biosynthesis
PWY.6147	-0.05117317926	0.02120566006	0.1783898825	6-hydroxymethyl-dihydropterin diphosphate biosynthesis I	Cofactor-Biosynthesis
PYRIDNUCSYN.PWY	-0.03947295541	0.01470717029	0.1756881841	NAD biosynthesis I (from aspartate)	Cofactor-Biosynthesis
PWY.6892	-0.05862628088	0.00211127126	0.08203796894	thiazole biosynthesis I (E. coli)	Cofactor-Biosynthesis
THISYN.PWY	-0.05608300397	0.009683246067	0.1410987284	superpathway of thiamin diphosphate biosynthesis I	Cofactor-Biosynthesis
PANTO.PWY	-0.05040195979	0.01264524329	0.1657219763	phosphopantothenate biosynthesis I	Cofactor-Biosynthesis
RIBOSYN2.PWY	-0.04491734943	0.000982103555	0.05161070219	flavin biosynthesis I (bacteria and plants)	Cofactor-Biosynthesis
P108.PWY	-0.05611345943	0.01687656697	0.1756881841	pyruvate fermentation to propanoate I	Fermentation
NAGLIPASYN.PWY	-0.06151006075	0.03159354502	0.2130606011	lipid IVA biosynthesis	Lipid-Biosynthesis
PWY.6467	-0.06110900162	0.03241046356	0.2150157583	Kdo transfer to lipid IVA III (Chlamydia)	Lipid-Biosynthesis
PWY.6703	-0.05637295488	0.02631912634	0.1983177847	preQ0 biosynthesis	SECONDARY-METABOLITE-BIOSYNTHESIS
PWY.6507	-0.04562301245	0.02790938226	0.1983177847	4-deoxy-L-threo-hex-4-enopyranuronate degradation	SECONDARY-METABOLITE-DEGRADATION
GLUCUROCAT.PWY	-0.05970682386	0.005041720407	0.09700305131	superpathway of β-D-glucuronide and D-glucuronate degradation	SECONDARY-METABOLITE-DEGRADATION
GOLPDLCAT.PWY	-0.1769427398	0.0149836236	0.1756881841	superpathway of glycerol degradation to 1,3-propanediol	Super-Pathways
PWY.6901	-0.06323344154	0.02041669144	0.1756881841	superpathway of glucose and xylose degradation	Super-Pathways
PWY.6897	-0.06270223199	0.001512684015	0.0726088327	thiamin salvage II	Super-Pathways
PWY.6467	-0.06110900162	0.03241046356	0.2150157583	Kdo transfer to lipid IVA III (Chlamydia)	Super-Pathways
GLUCUROCAT.PWY	-0.05970682386	0.005041720407	0.09700305131	superpathway of β-D-glucuronide and D-glucuronate degradation	Super-Pathways
THISYN.PWY	-0.05608300397	0.009683246067	0.1410987284	superpathway of thiamin diphosphate biosynthesis I	Super-Pathways
GALACT.GLUCUROCAT.PWY	-0.05352623777	0.01389390655	0.1756881841	superpathway of hexuronide and hexuronate degradation	Super-Pathways
PWY.7323	-0.04961117381	0.01961060837	0.1756881841	superpathway of GDP-mannose-derived O-antigen building blocks biosynthesis	Super-Pathways
COLANSYN.PWY	-0.04415063059	0.01751043647	0.1756881841	colanic acid building blocks biosynthesis	Super-Pathways
PANTOSYN.PWY	-0.04086930306	0.004019143793	0.09595114474	pantothenate and coenzyme A biosynthesis I	Super-Pathways
PWY.5913	-0.08105625381	0.03842318749	0.2322468221	TCA cycle VI (obligate autotrophs)	TCA-VARIANTS
PWY.6123	0.01903853739	0.0249823896	0.1979187371	inosine-5'-phosphate biosynthesis I	Nucleotide-Biosynthesis
PWY.7663	0.02001715026	0.03662202527	0.2290113368	gondoate biosynthesis (anaerobic)	Lipid-Biosynthesis

PWY.5973	0.0227374266	0.02998662897	0.2073651631	cis-vaccenate biosynthesis	Lipid-Biosynthesis
HISDEG.PWY	0.1216791687	0.03668680945	0.2290113368	L-histidine degradation I	Amino-Acid-Degradation
BIOTIN.BIOSYNTHESIS.PWY	0.319047977	0.01976728007	0.1756881841	biotin biosynthesis I	Super-Pathways
BIOTIN.BIOSYNTHESIS.PWY	0.319047977	0.01976728007	0.1756881841	biotin biosynthesis I	Cofactor-Biosynthesis
PWY.6519	0.3235450381	0.01984105858	0.1756881841	8-amino-7-oxononanoate biosynthesis I	Cofactor-Biosynthesis
PWY.5971	0.3269762344	0.01929039388	0.1756881841	palmitate biosynthesis II (bacteria and plants)	Lipid-Biosynthesis
PWYG.321	0.3296549682	0.02014096333	0.1756881841	mycolate biosynthesis	Lipid-Biosynthesis
PWY.7664	0.3301938468	0.02006855063	0.1756881841	oleate biosynthesis IV (anaerobic)	Lipid-Biosynthesis
PWY0.862	0.3307684069	0.0202318048	0.1756881841	(5Z)-dodec-5-enoate biosynthesis	Lipid-Biosynthesis
PWY.6282	0.3310216316	0.02035180229	0.1756881841	palmitoleate biosynthesis I (from (5Z)-dodec-5-enoate)	Lipid-Biosynthesis
PWY.5989	0.3310964705	0.02052118987	0.1756881841	stearate biosynthesis II (bacteria and plants)	Lipid-Biosynthesis
FASYN.INITIAL.PWY	0.3314080798	0.02038061956	0.1756881841	superpathway of fatty acid biosynthesis initiation (E. coli)	Super-Pathways
FASYN.INITIAL.PWY	0.3314080798	0.02038061956	0.1756881841	superpathway of fatty acid biosynthesis initiation (E. coli)	Lipid-Biosynthesis
P105.PWY	0.4834897532	0.03732660268	0.2290113368	TCA cycle IV (2-oxoglutarate decarboxylase)	TCA-VARIANTS
